# A rare diagnosis during evaluation for metabolic dysfunction associated steatotic liver disease: Glycogen storage disease type Ia presenting during adolescence in a male with severe obesity

**DOI:** 10.1002/jpr3.70247

**Published:** 2026-10-23

**Authors:** Carolena Trocchia, Rachel Herdes, Jodi Gedallovich, Amrita Narang

**Affiliations:** ^1^ Stanford Medicine Pediatric Gastroenterology Hepatology and Nutrition Palo Alto California USA; ^2^ Stanford Medicine Pathology Palo Alto California USA

**Keywords:** fatty liver disease, GLP‐1, hepatic mass, hepatomegaly, semaglutide

## Abstract

Metabolic dysfunction‐associated steatotic liver disease (MASLD) is a common comorbidity of pediatric obesity that predisposes patients to hepatocellular adenomas (HCAs). HCAs however are nonspecific to MASLD and warrant further evaluation. We present a case of a 16‐year‐old male with severe obesity, and persistently elevated liver enzymes, initially evaluated for MASLD. Imaging revealed multiple hepatic lesions consistent with HCAs, prompting further genetic evaluation. Trio exome sequencing identified biallelic variants in the glucose‐6‐phophatase gene, confirming a diagnosis of glycogen storage disease type Ia (GSD Ia). This case highlights the diagnostic challenge posed by overlapping features of MASLD and GSD Ia, particularly hepatic steatosis, HCAs, and metabolic syndrome. Initial management of MASLD with glucagon‐like peptide‐1 therapy was discontinued given risk for hypoglycemia, and the patient continues glucose monitoring and cornstarch therapy. This case emphasizes the importance of including inborn errors of metabolism in the differential diagnosis for multifocal HCAs and concerns with obesity treatments.

## INTRODUCTION

Metabolic dysfunction‐associated steatotic liver disease (MASLD) is a comorbidity of pediatric obesity with a rising prevalence of 7%–14% of children and adolescents in the United States.^1^, ^2^ Current diagnostic evaluation includes liver enzyme values, radiologic imaging, and/or liver biopsy. MASLD is a predisposing factor to both benign and malignant liver lesions.^3^ Hepatocellular adenomas (HCAs) are benign tumors with an incidence of 0.13 in 100,000 frequently associated with genetic disorders and pre‐existing liver abnormalities in pediatric populations with the potential for malignant transformation dependent on histologic type.^4^ These tumors are seen in individuals with obesity and metabolic syndrome, particularly among post‐pubertal females using oral contraceptives (who have a uniquely elevated incidence of 3–4 per 100,000).^4^ Males with hepatic adenomas require routine monitoring given risk for malignant transformation and hemorrhage.^4^

Currently, there are no pediatric‐specific guidelines for the classification and management of pediatric HCAs. In a comprehensive literature review of over 300 pediatric patients with HCAs, 84% had a known underlying genetic disorder—most commonly glycogen storage disease (GSD), with type Ia accounting for 45% of cases.^5^ A single‐center retrospective review of patients with GSD, 44% developed hepatic adenoma at a median age of 14.3.^5^

Given this strong association of HCAs and GSD, incidental findings of HCAs, especially multifocal lesions in males, should prompt further investigation into the underlying etiology and guide surveillance.^6^ GSD type Ia (GSD Ia) is an autosomal recessive disorder caused by mutations in the *glucose‐6‐phosphatase* (G6PC) gene, impairing glycogen breakdown and resulting in excessive hepatic glycogen accumulation and poor glucose regulation.^7^, ^8^ While typically diagnosed in infancy, delayed diagnoses have been documented in older children and adults.^8^

We present the case of an adolescent male with severe obesity who was found to have multiple HCAs during evaluation for MASLD. After extensive workup for hepatitis including infectious, metabolic, endocrine, autoimmune and oncologic etiologies, he was ultimately diagnosed with GSD Ia. This case underscores the importance of broadening the differential diagnosis in the context of hepatic adenomas, especially when encountered during MASLD evaluation.^6^

## CASE REPORT

A 16‐year‐old male with severe Class III obesity with body mass index (BMI) of 40.92 kg/m^2^, obstructive sleep apnea, hypertension, hypercholesterolemia with hypertriglyceridemia, and insulin resistance was referred to hepatology for persistently elevated alanine aminotransferase (ALT), which increased from 72 units per liter (U/L) to over 200 U/L. An initial abdominal ultrasound revealed hepatomegaly and hepatic steatosis. Although ALT levels temporarily improved following lifestyle modifications, they rebounded a year later (from 156 to 298 U/L), prompting a repeat ultrasound. This revealed at least five hypoechoic liver lesions, necessitating further evaluation with magnetic resonance imaging (MRI) and liver biopsy.

MRI of the abdomen with and without contrast demonstrated increased fat deposition and multiple hepatic lesions, the largest measuring 4.8 cm (Figure 1). Eovist contrast was used to evaluate the hepatobiliary phase and characterize these lesions with an enhancement pattern distinctive for HCAs, though one lesion appeared atypical. Targeted and background biopsies were obtained, which showed macrovesicular steatosis without fibrosis but no definitive features of adenoma (Figure 2A). Therefore, goal of management was directed on treatment of fatty liver disease using lifestyle modifications and initiation of glucagon‐like peptide‐1 (GLP‐1) medications given associated obesity, acanthosis nigricans, and co‐morbidities. ALT trended downward from 423 to 160 U/L following therapy, however with the MRI findings concerning for HCAs, a referral to genetics was made.

**Figure 1:**
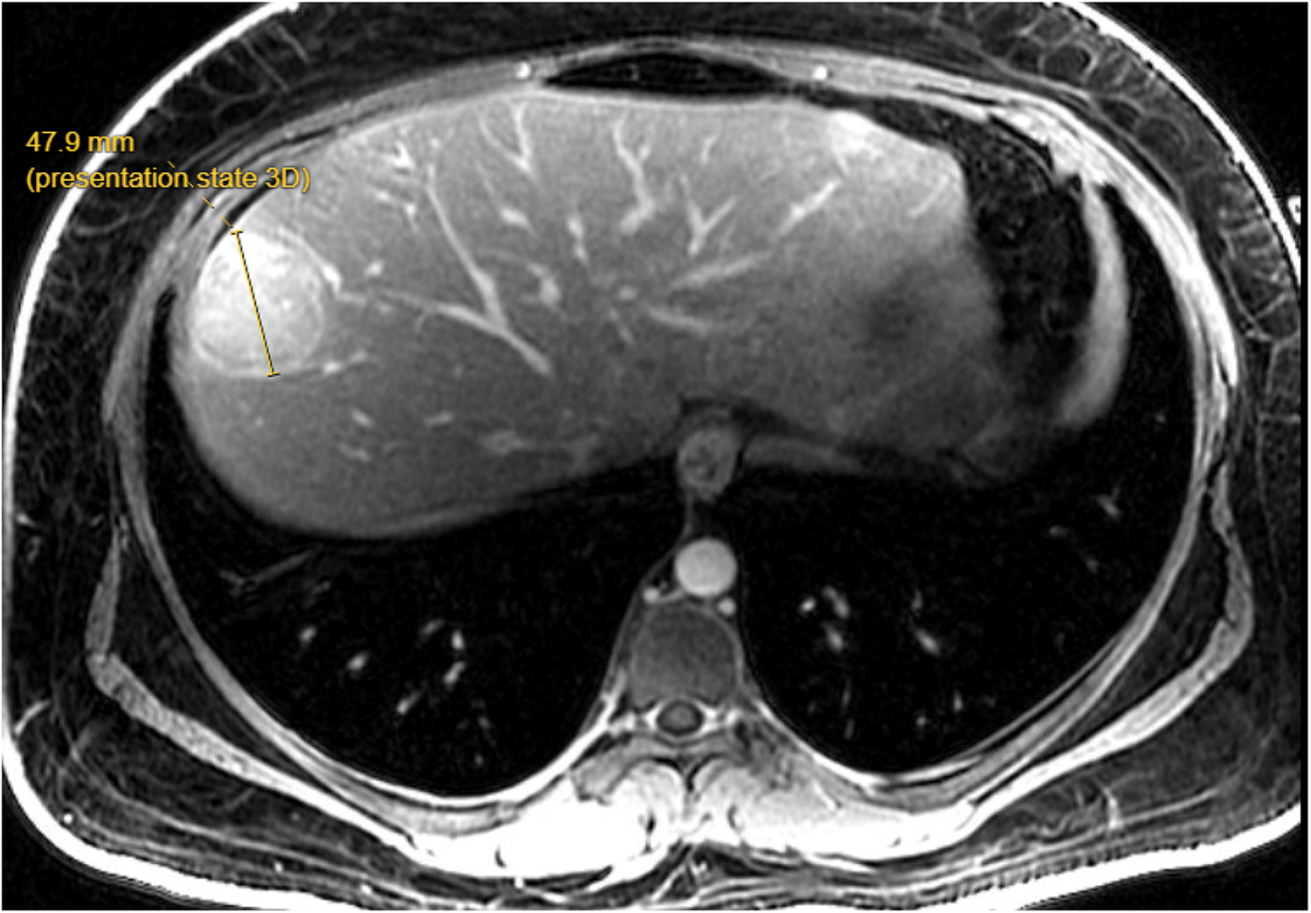
Abdominal MRI with IV Contrast Lava Flex sequence demonstrating the largest identified hepatic lesion 47.9 mm diameter. IV, Intravenous; MRI, magnetic resonance imaging.

**Figure 2:**
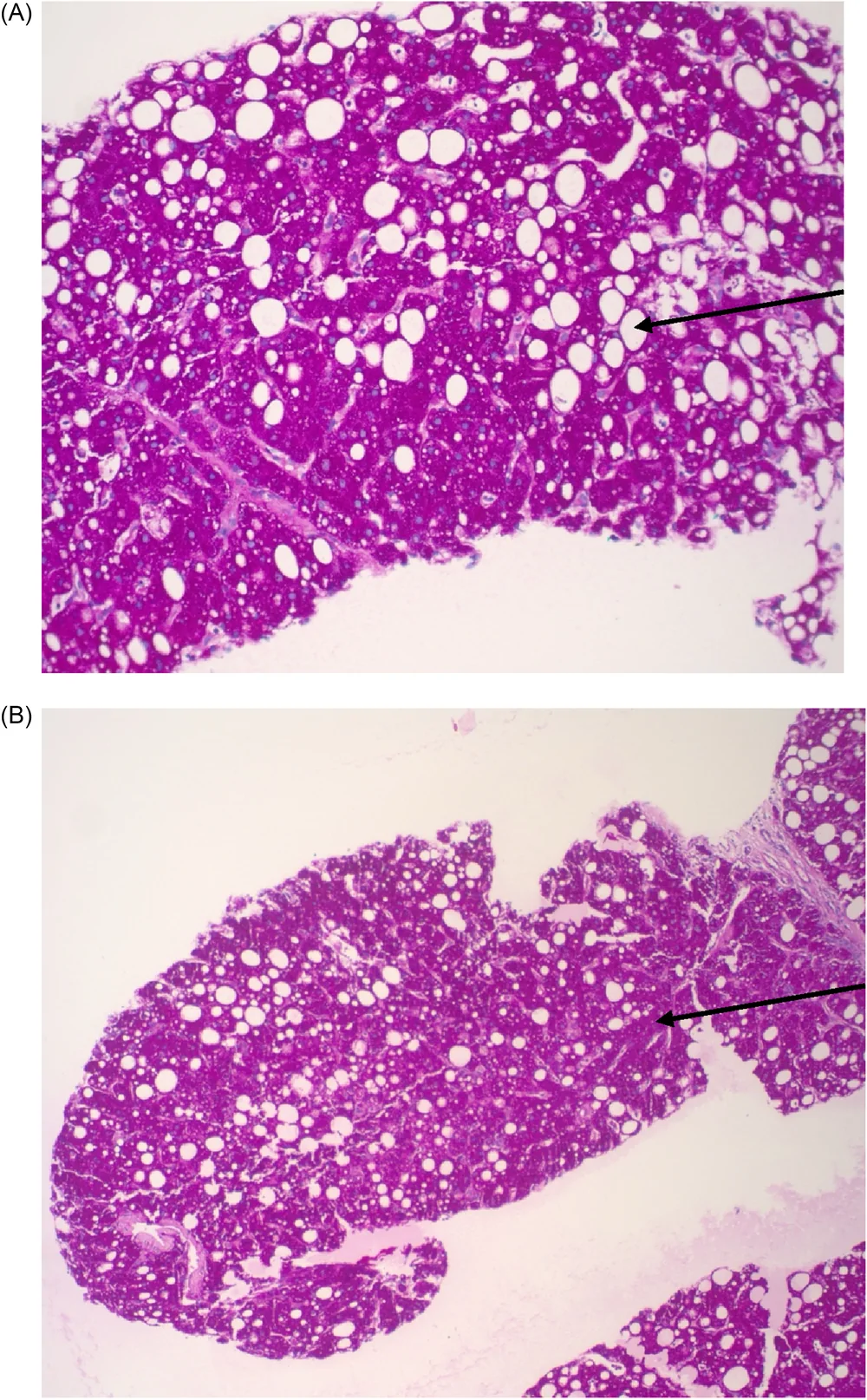
Histopathologic findings from liver biopsy taken from patients. (A) Liver biopsy demonstrating extensive steatosis with prominent fat globules (arrow) at ×20 magnification. (B) Periodic Acid–Schiff stain of liver biopsy subsequently performed on patient's liver biopsy (magenta color at arrow).

Upon genetics evaluation, he underwent trio exome sequencing which identified two biallelic variants within the G6PC gene associated with GSD1a: a maternal pathogenic variant (c.247 Cytosine > Thymine) and paternal variant of unknown significance (c.133 Guanine > Cytosine: p. Valine45Leucine). His workup further included the following elevated values: liver enzymes, uric acid, triglycerides and borderline low hemoglobin A1c with greater than 10 adenomas. He was subsequently diagnosed with GSD 1a and recommended to initiate uncooked cornstarch therapy (UCCS) with close glucose monitoring. He experienced episodes of hypoglycemia while taking semaglutide, which he used for approximately 8 months. Given this diagnosis, the decision was made to discontinue semaglutide for risk of hypoglycemia and delayed gastric emptying which may affect corn starch administration and efficacy.

## DISCUSSION

GSD Ia is a rare autosomal recessive disorder of glycogen metabolism, with an estimated incidence of 1 in 100,000 live births. It results from a deficiency in G6PC, disrupting the final steps of gluconeogenesis and glycogenolysis. This leads to glycogen accumulation, primarily in the liver and kidneys, and metabolic complications such as hypoglycemia, lactic acidosis, hyperlipidemia, and growth failure.^9^ Although GSD Ia typically presents in infancy with hypoglycemia and metabolic acidosis, delayed presentations may occur, often with hepatomegaly and signs mimicking Cushingoid features. Renal manifestations, such as nephrolithiasis, impaired renal function, and hypertension, is also common in GSD Ia. Other possible manifestations include fasting‐induced hypoglycemia, lactic acidosis, pancreatitis, pulmonary hypertension, and platelet dysfunction‐related bleeding diathesis.^9^

In this case, the patient (consent obtained) presented with several findings consistent with both GSD Ia and metabolic syndrome: hepatic steatosis, HCAs, dyslipidemia, and hypertension (File S1). These overlapping features complicate the diagnostic process and highlight the importance of considering inborn errors of metabolism in the differential diagnosis of HCAs in pediatric patients. Patients with overweight and/or obese BMI status age 5 or less should be considered for genetic and monogenic obesity testing (this patient had class II obesity at age 8, no prior history known).^10^

This case also underscores the potential limitations of biopsy and imaging in identifying hepatic adenomas and their underlying etiologies. Although imaging suggested HCAs, the initial biopsy was inconclusive—potentially due to sampling error. A complete list of pathology stains performed, along with an explanation for each stain, is provided in File S1. Notably, the initial biopsy staining panel did not include a Periodic Acid–Schiff (PAS) stain, as normal hepatocytes inherently contain PAS‐positive glycogen. Histologic confirmation of GSD Ia typically involves PAS staining, which highlights glycogen and glycoproteins with a magenta coloration (Figure 2B), followed by diastase digestion to cleave glycogen and eliminate the magenta staining, thereby confirming glycogen accumulation. PAS stain was later included (Figure 2B), however the family declined any repeat biopsy.^10^

Notably, conventional obesity treatments—such as caloric restriction and GLP‐1 receptor agonists—may pose significant risks in patients with GSD Ia by precipitating hypoglycemia. As a precaution, the patient's semaglutide therapy was therefore discontinued, and he was started on glucose monitoring with calorie controlled dietary therapy with cornstarch. There is no data establishing the use of GLP‐1 medications in pediatric patients with GSD 1a and HCAs, consequently their impact on serum glucose is not known. Further studies are required to better understand the role of GLP‐1 medications within this population while on dietary restrictions for GSD 1a treatment as most children with GSD will struggle with obesity with increased calories from UCCS.

## CONCLUSION

This case highlights the need for heightened clinical suspicion for metabolic disorders such as GSD Ia in adolescent males presenting with multifocal hepatic adenomas, even when findings are attributed to obesity‐related MASLD. Prompt recognition and diagnosis are critical to prevent complications including malignant transformation of HCAs and progressive renal disease. Moreover, caution is warranted when considering GLP‐1 therapy in this population due to the risk of hypoglycemia. Additional research is necessary to clarify the role and safety of such treatments in patients with GSD Ia.

## CONFLICT OF INTEREST STATEMENT

The authors declare no conflicts of interest.

## ETHICS STATEMENT

Patient consent was provided through the patient's parents with the intent to publish and agree to it.

## Supporting information

Supporting File 1.

## Data Availability

Data sharing is not applicable to this article as no datasets were generated or analyzed during the current study.

## References

[1] Stroes ASR , Vos M , Benninga MA , Koot BGP , Koot BGP . Pediatric MASLD: current understanding and practical approach. Eur J Pediatr. 2024;184(1):29. 10.1007/s00431-024-05848-1 39560782

[2] Takahashi Y , Dungubat E , Kusano H , Fukusato T . Pathology and pathogenesis of metabolic dysfunction‐associated steatotic liver disease‐associated hepatic tumors. Biomedicines. 2023;11(10):2761. 10.3390/biomedicines11102761 PMC10604511 37893134

[3] Kolbe AB , Acord MR , Khanna G , et al. Imaging findings and management strategies for liver masses in children with predisposition disorders: a review by the Pediatric LI‐RADS Group. Radiographics. 2025;45(1):e240063. 10.1148/rg.240063 39666572

[4] Espinoza AF , Vasudevan SA , Masand PM , Lòpez‐Terrada DH , Patel KR . Pediatric hepatocellular adenomas: what is known and what is new? Cancers. 2023;15(19):4790. 10.3390/cancers15194790 PMC10571754 37835484

[5] Jang HJ , Yang HR , Ko JS , Moon JS , Chang JY , Seo JK . Development of hepatocellular carcinoma in patients with glycogen storage disease: a single center retrospective study. J Korean Med Sci. 2020;35(1):e5. 10.3346/jkms.2020.35 PMC6942134 31898434

[6] Scheers I , Tambucci R , De Magnee C , et al. Paediatric hepatocellular adenomas: lessons from a systematic review of relevant literature. JHEP Rep. 2024;6(6):101078. 10.1016/j.jhepr.2024.101078 PMC11061330 38699071

[7] Xu Y , Liu Y , Yuan Y , et al. Clinical characteristics of 50 hepatocellular adenoma patients among 164 cases of glycogen storage disease type Ia. Eur J Pediatr. 2025;184(7):458. 10.1007/s00431-025-06180-y 40586975

[8] Nana M , Anastasopoulou C , Parikh NS , et al. Glycogen Storage Disease Type I [Internet]. StatPearls Publishing; 2025. https://www.ncbi.nlm.nih.gov/books/NBK534196/. 30480935

[9] Malhotra S , Sivasubramanian R , Srivastava G . Evaluation and management of early onset genetic obesity in childhood. J Pediatr Genet. 2021;10(3):194‐204. 10.1055/s-0041-1731035 PMC8416234 34504723

[10] Derks TGJ , Rodriguez‐Buritica DF , Ahmad A , et al. Glycogen storage disease type Ia: current management options, burden and unmet needs. Nutrients. 2021;13(11):3828. 10.3390/nu13113828 PMC8621617 34836082

