# Supplementary material for: A rare diagnosis during evaluation for metabolic dysfunction associated steatotic liver disease: Glycogen storage disease type Ia presenting during adolescence in a male with severe obesity
Source: JPGN Rep. 2026 Oct 23:10.1002/jpr3.70247. Online ahead of print. doi: 10.1002/jpr3.70247 (PMC13691576; doi:10.1002/jpr3.70247)
Supplement: Supplementary file 1 [file JPR3-9999-0-s001.docx]

**Immunohistochemical and Special Stains Summary used for work-up of hepatic masses and for Glycogen Storage Disease Type 1a (GSD 1a)**

**Stain Description**

**Amyloid A (AA):** Used to support the diagnosis of systemic amyloidosis by specifically staining for AA amyloid protein.

**Beta-Catenin:** Observed in certain subtypes of hepatocellular adenoma and hepatocellular carcinoma. Positivity indicates mutations involving the wingless-related integration (Wnt)-Beta catenin signaling pathway.

**Cluster of Differentiation 34 (CD34):** A transmembrane glycoprotein that serves as an endothelial marker identifying highly vascular tissue. Its expression supports differentiation of hepatocellular carcinoma from hepatic adenoma.

**C-Reactive Protein (CRP):** Increased expression is associated with inflammation and may be indicative of inflammatory hepatic adenomas.

**Glutamine Synthetase:** Displays distinct staining patterns in various hepatic lesions. For instance, a “map-like” pattern is characteristic of focal nodular hyperplasia, while diffuse staining is typically seen in hepatocellular carcinoma.

**Glypican-3:** Utilized to distinguish hepatocellular carcinoma from hepatic adenoma, as it is typically positive in malignant hepatocytes.

**Liver Fatty Acid Binding Protein (LFABP):** Loss of LFABP staining is suggestive of hepatocellular adenoma.

**Reticulin:** Highlights connective tissue framework. A reduction in reticulin staining is commonly observed in hepatocellular carcinoma.

**Trichrome Stain:** Used to assess collagen deposition, serving as a marker of hepatic fibrosis.

**Periodic Acid–Schiff (PAS) Stain: *(Not performed)***
This stain identifies cytoplasmic glycogen within hepatocytes and serves as a diagnostic tool for glycogen storage disease type Ia (GSD1a). Positive staining demonstrates a diffusely magenta cytoplasmic pattern throughout the liver parenchyma.
